# The Role of mTOR in Neuroendocrine Tumors: Future Cornerstone of a Winning Strategy?

**DOI:** 10.3390/ijms19030747

**Published:** 2018-03-06

**Authors:** Giuseppe Lamberti, Nicole Brighi, Ilaria Maggio, Lisa Manuzzi, Chiara Peterle, Valentina Ambrosini, Claudio Ricci, Riccardo Casadei, Davide Campana

**Affiliations:** 1 Department of Experimental, Diagnostic and Specialty Medicine, S.Orsola-Malpighi University Hospital, 40138 Bologna, Italy; lamberti.giu88@gmail.com (G.L.); nicolebrighi@hotmail.com (N.B.); ila.mag.88@gmail.com (I.M.); lisamanuzzi@gmail.com (L.M.); chiara.peterle@gmail.com (C.P.); 2 Nuclear Medicine Unit, Medicina Nucleare Metropolitana, S.Orsola-Malpighi University Hospital, 40138 Bologna, Italy; valentina.ambrosini@unibo.it; 3 Department of Medical and Surgical Sciences, S.Orsola-Malpighi University Hospital, 40138 Bologna, Italy; claudio.ricci6@unibo.it (C.R.); riccardo.casadei@unibo.it (R.C.)

**Keywords:** mTOR, neuroendocrine tumor, neuroendocrine tumors, everolimus, RAD001, RADIANT, mTORC1, PTEN, carcinoid, Akt

## Abstract

The mechanistic target of rapamycin (mTOR) is part of the phosphoinositide-3-kinase (PI3K)/protein kinase B (AkT)/mTOR pathway and owes its name to the inhibitory effect of rapamycin. The mTOR has a central converging role for many cell functions, serving as a sensor for extracellular signals from energy status and nutrients availability, growth factors, oxygen and stress. Thus, it also modulates switch to anabolic processes (protein and lipid synthesis) and autophagy, in order to regulate cell growth and proliferation. Given its functions in the cell, its deregulation is implicated in many human diseases, including cancer. Its predominant role in tumorigenesis and progression of neuroendocrine tumors (NETs), in particular, has been demonstrated in preclinical studies and late clinical trials. mTOR inhibition by everolimus is an established therapeutic target in NETs, but there are no identified predictive or prognostic factors. This review is focused on the role of mTOR and everolimus in NETs, from preclinical studies to major clinical trials, and future perspectives involving mTOR in the treatment of NETs.

## 1. Introduction

Neuroendocrine tumors (NETs) originate from neuroendocrine cells scattered throughout the body. Classification of NETs is based on primary site of origin (bronchopulmonary system, gastrointestinal tract and pancreas are the most frequent), proliferation index (according to ki67) and symptoms from production of biologically active amines (functioning vs. non-functioning NET). Their incidence is raising, possibly due to greater awareness of this rare disease (5.25/100.000/year) [1], increased detection and a real increase in the number of cases/year [2]. Although NETs from different primary sites have different prognosis and behaviour, they have been shown to share key biological features. This can explain effectiveness of the same treatment, such as *somatostatin analogs* (SSA) or everolimus, across different types of NET. Everolimus (former RAD001, Novartis), is a potent and selective inhibitor of the mechanistic target of rapamycin (mTOR). This is a key downstream member of the phosphoinositide-3-kinase (PI3K)/protein kinase B (AkT)/mTOR pathway [3], which has a central role in regulation of cell cycle in response to environmental stimuli, such as available energetics and stress. This pathway is often altered in many types of cancers [4]: hormone-resistant breast cancer [5], clear cell renal cell carcinoma [6] and NETs [7,8,9].

The paramount role of mTOR in NETs was highlighted by the RAD001 in Advanced Neuendocrine Tumors (RADIANT) series of clinical trials. In these studies, everolimus proved to be effective in lung, pancreatic, gastroenteric and unknown/other origin well-differentiated/low-grade NETs.

This review presents current understanding of the role of mTOR in NET tumorigenesis, beginning from mTOR functions in normal cell and common deregulation events occurring in cancer. The review also focuses on present implications and perspectives for therapy of this rare type of cancer.

## 2. mTOR in the Normal Cell

**Figure 1:**
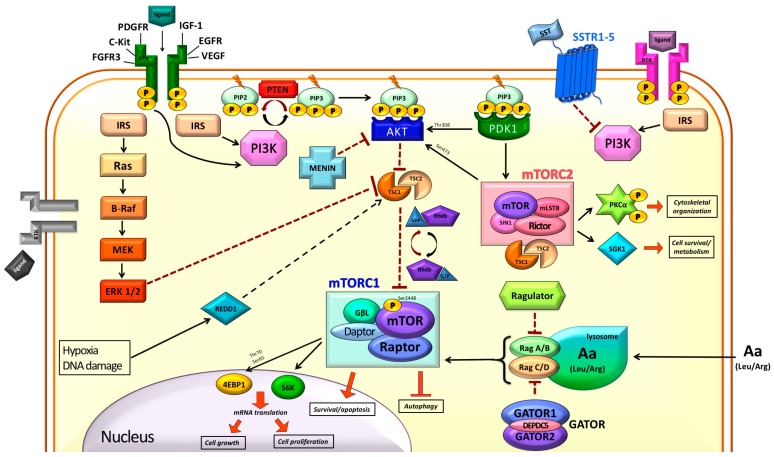
Schematic representation of the phosphoinositide-3-kinase (PI3K)/protein kinase B (AkT)/ mechanistic target of rapamycin (mTOR) pathway. Black arrow: direct activation; Dotted black arrow: activation through unknown/not diasplayed other molecules; Dotted T bar: inhibition; Black/red twisting arrows: activation/inactivation conversion; Red arrow: promotion of processes; Red T bar: inhibition of processes; P in yellow spheres: phosphate group.

mTOR is a conserved serine/threonine protein kinase and is a member of the family of the PI3K-related kinases [10]. As the name itself suggests, mTOR is inhibited by rapamycin (or sirolimus), a macrolide produced by *Streptomyces Hygroscopicus*, named after *Rapa Nui* (native language for Easter Island). The PI3K/Akt/mTOR pathway had been extensively studied and found to be more involved than expected (Figure 1).

mTOR acts as the catalytic subunit of two large complexes, mTOR complex 1 (mTORC1) and mTOR complex 2 (mTORC2). These complexes have different upstream and downstream interactions, reflecting distinct roles in regulation of cell functions. They also show different sensitivity to rapamycin: mTORC1 is rapidly inhibited by rapamycin, while mTORC2 inhibition needs longer exposure [3,11,12].

Besides mTOR, the regulatory-associated protein of mammalian target of rapamycin (raptor) is a key component of mTORC1 [12]. The mTORC1 serves as an integrate sensor of energy status and nutrients (amino acids) [12], growth factors [13,14], oxygen and stress [15,16,17] and its activation status regulates major cell processes such as protein and lipid synthesis (promoting anabolism) and autophagy.

The mTORC2 is composed of mTOR and the rapamycin-insensitive companion of mammalian target of rapamycin (rictor), together with other proteins [18]. Contrary to what was previously thought, longer exposure to rapamycin eventually inhibits mTORC2 activity as well, by interfering with its assembly [11]. In general, mTORC1 role is better defined than that of mTORC2, which is considered a well distinct branch of the PI3K/Akt/mTOR pathway.

### 2.1. Modulation of mTOR Activity

Growth factors transmit signals by means of their receptor tyrosine-kinase (RTK) which recruits PI3K to the cytoplasmic membrane and activate it. The PI3K then converts phospatidylinositol-4,5,-bisphospate (PIP2) to phospatidylinositol-3,4,5,-trisphospate (PIP3) by phosphorylation [14]. Akt binds PIP3 by its Pleckstrin-homology domain (PH) eventually resulting in mTORC1 activation. The phosphatase and tensin homolog deleted on chromosome 10 (PTEN) removes the phosphate from PIP3 to obtain again PIP2, unable to activate Akt, and acts as a negative regulator of the pathway [19].

Signals from RTKs, via extracellular-signal-regulated kinase 1/2 (ERK1/2), ribosomal S6 kinase (RSK1) and Akt, are forwarded to mTORC1 through tuberous sclerosis 1/2 (TSC1/2) inhibition, eventually resulting in activation of the pathway as well [20,21]. In a similar way, pro-inflammatory cytokines (e.g., tumor necrosis factor alfa (TNFa)) and wingless-related integration site (Wnt) canonical pathway can activate mTORC1 through TSC1/2 inhibition [22,23]. However, mTORC1 activity can also be enhanced by Akt in a TSC1/2-independent manner, via the proline-rich Akt-substrate of 40KDa (PRAS40) [24].

mTOR relays signals from several RTKs and pathways converging on mTORC1, namely insulin receptor, insulin-like growth factor 1 (IGF-1) and its receptor (IGF-1R), epidermal growth factor receptor (EGFR), vascular endothelial growth factor receptor (VEGFR), platelet-derived growth factor receptor (PDGFR), fibroblast growth factor receptor-3 (FGFR3), c-Kit and somatostatin receptors 1-5 (SSTR1-5) [14,25,26,27,28].

The guanosine triphosphate (GTP)-bound Ras homolog enriched in brain (Rheb) is a small G-protein which promotes mTOR kinase activity by mechanism which is not fully understood. Upstream negative regulation of mTORC1 depends on the TSC1/TSC2 heterodimer: it functions as a GTPase activating protein (GAP) towards Rheb and stimulates its intrinsic GTP-ase activity, hampering mTOR activation [3,29].

Stress signals enhance TSC1/2 inhibitory effect on Rheb, thus resulting in downregulation of the pathway: adenosine monophosphate-activated protein kinases (AMPK) phosphorylate TSC2 in the presence of low energy and hypoxia [15], while DNA damage cause p53-dependent increase of transcription of *TSC2* and *PTEN* genes, downregulating the whole PI3K pathway [16,17]. Low oxygen tension and DNA damage also induces the expression of the DNA damage response (REDD1) which leads to TSC2 activation and eventually to the lowering of mTORC1 signaling. REDD1 was thought to sequestrate 14-3-3 proteins, that bind and inhibit TSC2 [30]. However later findings have questioned REDD binding to 14-3-3 [31].

On the other hand, amino acids, mainly leucine and arginine, can stimulate mTORC1 activity in an Akt-independent manner, via Ras-related GTPases (Rag) and the Ragulator complex [32,33,34]. In the presence of amino acids sufficiency, GTP-bound Rag interacts with raptor and promotes the translocation of mTORC1 to the lysosomal membrane, where Rag binds the Ragulator complex, and its activation. The interaction between Rag and Ragulator is essential for the activation of mTORC1 by amino acids. A complex with GAP activity towards Rag was identified and named GATOR. It is made of two sub complexes, GATOR1 and GATOR2. GATOR1 in turn is made of 3 subunits (DEPDC5, Nprl2 e Nprl3) and GATOR2 is made of 5 subunits (Mios, WDR24, WDR59, Seh1L, Sec13) defining GATOR as an octameric complex. In case of amino acids starvation, GATOR 1 exerts its GAP activity on RagA and RagB, eventually reducing mTORC1 signaling. Furthermore, GATOR2 negatively regulates GATOR1, through interaction with its DEP domain containing 5 (DEPDC5) subunit [35].

Intra-pathway modulation of the activity occurs via negative feedback loops. Inhibition of mTORC1 determines a consequent increase in Akt activity, due to upregulation of RTK/IRS-1 signals [36,37] possibly via phosphorylation on Akt-Ser473 by mTORC2 [18] or direct phosphorylation on Akt-Thr308 by the 3-phosphoinositide-dependent kinase-1 (PDK-1). Phosphorylation of Akt on Ser473 determines its maximal activation, while phosphorylation on Thr308 is sufficient to drive cell survival [38]. The mitogen-activated pathway kinase (MAPK) pathway signaling, through S6 kinase (S6K) activation, is also enhanced by mTORC1 inhibition [39].

In response to growth factors and their receptors, but not nutrients, briefly, mTORC2 activates three AGC subfamily kinases: Akt, serum- and glucocorticoid-induced protein kinase 1 (SGK1) and protein kinase C-alfa (PKCa). It is tempting to speculate that a role of mTORC2 would be to promote cell proliferation in the presence of growth factors, irrespective of nutrients availability.

The phosphorylation of Akt on Ser473 by mTORC2, locates the mTOR both upstream (mTORC2) and downstream (mTORC1) of Akt itself [3]. Through the activation of GSK1, mTORC2 is involved in cell growth and ion transport [40], while through PKCa it regulates cytoskeleton organization and cell shape [41,42].

### 2.2. Downstream of mTORC1

As part of the mTORC1 complex, mTOR directly phosphorylates the translational regulators S6K1/2 on Thr-389, and the eukaryotic translation initiation factor 4E binding protein 1/2 (4EBP1/2) [3]. This increases mRNAs translation and protein synthesis and, ultimately, growth and proliferation of the cell [43,44]. In a series of lung NET-derived cell lines [45], S6K activation is found to better correlate with mTOR activation in respect to 4EBP1. This finding may be explained by the fact that 4EBP1 has several phosphorylation sites [46,47], which have different sensitivity to rapamycin and are the target of other kinases as well [48,49].

For the purpose of facilitating cell growth and proliferation, mTORC1 also promotes synthesis of lipids involved in new membrane production and increases cellular metabolism. Lipids production is mainly regulated through the sterol regulatory element-binding protein 1/2 (SREBP1/2) [50,51] and by promoting the expression of the master regulator of adipogenesis, the peroxisome proliferator-activated receptor gamma (PPAR-y) [52,53]. Positive regulation of cellular metabolism is achieved via hypoxia inducible factor 1alfa (HIF1a): it increases glycolytic activity through promotion of transcription and translation of several glycolytic genes [50,54,55].

In addition to positive regulation of anabolic processes, mTORC1 is also an inhibitor of cellular catabolism. Autophagy, the main adaptation mechanism to nutrient starvation, and lysosome biogenesis, are both designated to cell degradation and components recycling, and both are negatively modulated by mTORC1 in response to nutrient availability [13,56,57,58].

## 3. mTOR and Cancer

The key role of mTOR in cell growth and energetics and inhibition of autophagy suggests how the pathological activation of this pathway is bound to cell survival, proliferation and cancer development [27]. So it is unsurprising that PI3K/Akt/mTOR pathway is the second most mutated one in cancer [4], after p53. Notably, p53 loss has also been found to lead to activation of mTORC1 [16].

Downstream to mTORC1, 4EBP1 has shown to be the main effector of the oncogenic signaling [59]. The eukaryotic translation initiation factor 4E (eIF4E) released by hierarchical phosphorylation of 4EBP1 [46], promotes translation of several genes involved in processes crucial for tumorigenesis: protein synthesis, cell survival and proliferation, neoangiogenesis and glycolysis. Neoangiogenesis and glycolysis are mediated by HIF1a [13]. Moreover, mTORC1 activation promotes tumorigenesis through the activation of SREBP1, essential for the synthesis of lipids for membrane assembling and turnover [50] and for the inhibition of autophagy. The role of autophagy in cancer, however, has to be better defined [60].

Due to several cross-links with other pathways, secondary activation of mTOR and its pathway has also been identified as a mechanism of resistance or escape from cancer therapy, for example, in breast cancer hormone therapy [61]. Everolimus plus exemestane (a steroidal aromatase inhibitor) was approved in hormone-receptor-positive advanced breast cancer that had recurrence or progression while receiving previous therapy with a nonsteroidal aromatase inhibitor [5]. Recently, hyperactivation of PI3K through PTEN loss has been linked to impaired T-cell priming, causing a non-T-cell inflamed tumor microenvironment in melanoma [62]. PTEN loss by mutations or deletions may occur as an acquired immune escape mechanism from immunotherapy. The exact mechanism is still unclear, but could involve the increased expression of vascular endothelial growth factor (VEGF) and/or the inhibition of autophagy mediated by PI3K pathway activation [63].

## 4. mTOR and NETs

The deregulation of the PI3K/Akt/mTOR pathway has a well-established role in NETs that is supported by many findings, lastly by the clinical efficacy shown by the rapamycin analog (rapalog), everolimus.

Studies on preclinical models of panNETs (e.g., RIP1-Tag2 oncomice) showed that activating alterations of mTOR were specific of tumor cells, when compared to normal tissue, and that treatment with rapamycin reduced proliferation and increased apoptosis. Interestingly, in vivo studies showed that tumors had increased mTOR signaling after progression to rapamycin, suggesting the presence of an escape mechanism which was prevented by contemporary treatment with rapamycin and erlotinib, an EGFR inhibitor [21].

### 4.1. Genetic Alterations

NETs are part of the clinical manifestations of some rare familial syndromes with variable penetration. These syndromes are caused by mutations in genes variably linked to the PI3K/Akt/mTOR pathway, such as Multiple Endocrine Neoplasia-1 (*MEN1*) [64,65], Neurofibromatosis-1 (*NF1*) [66,67,68,69], Tuberous sclerosis (*TSC1/2*) [70,71,72], Von Hippel-Lindau (*VHL*) [65,73] and Cowden syndrome (*PTEN*) [74,75,76].

Loss of heterozygosity in the *NF1* gene is associated with activation of mTOR signaling [70] and mutations of this gene are found in 1–2% of sporadic NETs [77].

Sporadic pancreatic NETs (panNETs) have been showed to harbor activating mutations of the mTOR pathway as well. This was observed in about 15% of cases, namely on PTEN and TSC2 [8]. In a more recent and wider series of panNETs, whole-genome sequencing was performed on samples from 102 primary panNETs. In this work, Scarpa et al. identified somatic driver mutations occurring on genes already known to be related to NETs development, such as *MEN1* (37% of tumors) and mutually exclusive inactivating mutations of *DAXX* and *ATRX* (22% and 11%, respectively). Somatic driver mutations were also observed in genes of the PI3K/Akt/mTOR pathway in about 13% of samples: *PTEN* was mutated in 7 samples, while *TSC1* and *TSC2* genes were mutated in 2 samples each [78]. Interestingly, 2 other samples harbored mutations in the *DEPDC5* gene, which encodes for a subunit of the negative regulator of mTORC1 activity, GATOR1 [35]. Inactivating mutations on negative regulators of the mTOR pathway (i.e., *PTEN*, *TSC1*, *TSC2* and *DEPDC5*) were mutually exclusive, strengthening their role of driver mutations in panNETs. Previously undescribed genetic alterations that lead to secondary mTOR activation were also described. Ewing Sarcoma Breakpoint Region 1 (*EWSR1*) was involved in gain-of-function fusion events in 3% of the examined panNETs: in two cases with the *BEND2* gene and in one case with the *FLI-1* gene. Furthermore, the 19p chromosome region containing the Persephin gene (*PSPN*) was amplified in 13% of samples [78]: PSPN binds the rearranged during transfection (RET) receptor and activates PI3K catalytic subunit alfa (PI3KCA) [79].

DNA from 54 primary lung NETs, including low grade (17 typical carcinoids (TCs) and 8 atypical carcinoids (ACs)) and high grade NETs (17 large cell neuroendocrine carcinomas and 12 small cell neuroendocrine carcinomas), was sequenced. Five missense mutations in *mTOR* (*n* = 1), *TSC1* (*n* = 1) and *TSC2* genes (*n* = 3) were identified, but only in low grade tumors [80].

*MEN1* gene is mutated in over 40% of sporadic panNETs [8,78] and regions involving MEN1 gene (located on the 11q13.1) are lost in 70% of sporadic panNETs [78]. Menin is the product of the *MEN1* gene and acts as a tumor-suppressor; however, the exact mechanism of action is not completely understood. Menin, nevertheless, has also been observed to be a negative regulator of Akt by binding and sequestering it to the cytoplasm, preventing its migration to the plasma membrane and its activation. This function of menin is lost by *MEN1* mutated gene, in particular when the *W436R* mutation occurs [81].

### 4.2. Expression Studies

Genetic alterations in mTOR pathway genes seldom reflect altered expression of their products in NETs. Since immunohistochemistry (IHC) is not expensive and has a wide diffusion and high reproducibility, it has been used to correlate the expression of key proteins in NETs with outcome and response to treatments, in order to identify prognostic and predictive factors, respectively [82].

Studies on cell lines from small bowel NETs derived from primary tumours, liver metastases and metastatic lymph nodes, highlighted the strong PI3K/Akt/mTOR pathway activation in respect to normal enterochromaffin-like cells [18].

In NETs of various origins, including lung, stomach, duodenum, small bowel, appendix, and colon, mTOR expression was observed to be significantly higher in primary lesions than in metastases [83]. This finding is coherent with a driver role of mTOR pathway activation in NETs tumorigenesis, suggesting that other alterations occur upon progression or development of metastatic disease. Metastases then may be independent on mTOR.

An Italian study on mTOR pathway in lung NETs highlighted the activation of mTOR (by means of IHC for Ser2448 phosphorilated-mTOR, p-mTOR) and pS6K in TCs and ACs. Positivity for p-mTOR was related to prognostically favorable characteristic (negative lymph nodes in ACs, increased disease-free survival after radical resection). However, no improved overall survival was observed in p-mTOR-positive tumors, neither in low grade nor high-grade lung NETs [45]. A similar result was observed in another series of 42 panNETs [84].

In our series of 64 radically-removed gastro-enteropancreatic (GEP) NETs, the prognostic role of p-mTOR(Ser2448), SSTR2A and IGF-1R was explored. We observed that higher p-mTOR IHC staining was a negative prognostic factor since it correlated with more advanced stage at diagnosis, a shorter disease-free survival and a higher risk of relapse after radical resection [85]. In another series, high p-mTOR expression has been found with a higher rate in large cell GEP-NETs than in lower-grade NETs, suggesting an association between mTOR activation and an aggressive biological behavior [86].

Thirty-seven patients affected by neuroendocrine carcinomas (NEC) were enrolled in a phase II clinical trial on temsirolimus, an intravenous rapalog. In 13 paired biopsies (before and after treatment), higher pre-treatment p-mTOR(Ser2448) staining was associated with a better response to the drug. Furthermore, an increase in p-Akt(Ser473) and a decrease in p-mTOR after two weeks of treatment were associated with a better outcome. The increase in p-AKT in response to mTOR inhibition may be due to the presence of a feedback loop through mTORC2 and/or RTKs (namely IGF-1R) [38,87].

PTEN expression is lost in 7–29% of panNETs, while conserved in normal pancreatic islet cells [7,78,88]. In a series of 72 panNETs, PTEN expression was low or absent in 60% of cases. Low/absent PTEN correlated to worse prognosis (disease-free, progression-free and overall survival). Similarly, TSC2, was highly expressed in normal islet cells but lost in up to 35% of specimens. PanNETs lacking of TSC2 expression showed a more aggressive biological behavior and worse prognosis [7].

Results from the aforementioned studies were contrasting, thus preventing us from the identification of a reliable predictive or prognostic factor.

### 4.3. Effects of mTOR Inhibition

Everolimus significantly reduced viability in cell lines derived from small bowel NETs (primary tumours and liver metastases). The effect was increased by adding octreotide. Everolimus was also effective in reducing p-mTOR (Ser2448) in this model. However, viability increased again after 72 h of exposition to the drug, suggesting establishment of an escape mechanism, possibly through an intra-pathway feedback or through cross-activation from other pathways, such as RTKs. Occurrence of escape did not seem to be corrected by adding the SSA octreotide [18]. The involvement of RTKs was also highlighted in the research by Chiu et al., who studied effects on proliferation and apoptosis in panNETs in Rip1-TAG2 mice when treated with rapamycin, erlotinib or both. With the combination of rapamycin and erlotinib, more marked increase in apoptosis and more profound decrease in ki67 than with each single drug were observed. Furthermore, interestingly, the authors reported a decrease in pAkt (Ser473) as well in Beta-TC3 cell lines when exposed to the combination of everolimus and erlotinib [21]. Similarly, everolimus showed a significant antiproliferative effect in the rat-derived insulinoma cell line, INS1, that was increased by adding octreotide. The p-mTOR (Ser2448) and pS6K were similarly significantly decreased, but the magnitude of the effect was not amplified by the combination with octreotide [89].

Commercial cell lines of TCs and ACs (i.e., H727 and H720, respectively) showed different sensitivity to everolimus. In particular, when exposed to everolimus, sensitive cell lines, that is, H720, showed an increase in pS6K, but a decrease in pAkt (Ser473). On the contrary, insensitive cell lines, namely H727, showed and inverse pattern, this being indirect evidence of the presence of a negative feedback loop between mTORC1 and Akt. In this paper, no correlation between p-mTOR (Ser2448) and response to drug was observed [45]. A similar pattern was observed in panNETs: exposure of cell lines to rapamyicin and everolimus induced reduction in phosphorylation of S6K and 4EBP1, with a secondary increase in pAkt (Ser473) [7,90]. This data about a negative feedback loop within the mTOR pathway, suggested the rationale for using a combined inhibition of targets, by multi-target inhibitors or by combinations of drugs.

Given the prominent clinical role of everolimus, the identification of a predictive factor of response to mTOR inhibition would be of paramount importance in order to select the best therapeutic option to be proposed to patients. In a study on cultured cells from 24 lung NETs, mostly TCs, higher expression of mTOR and p-mTOR (Ser2448) was associated to response to everolimus in vitro [91]. However, no clinical predictive marker has been identified so far.

## 5. Clinical Trials

Clinical activity and efficacy of everolimus was evaluated in the RAD001 in Advanced Neuendocrine Tumors (RADIANT) saga of trials (Table 1). The first one of this series of trials, the RADIANT-1, was an international open-label phase II trial lead in 160 patients affected by panNETs progressing after previous cytotoxic chemotherapy. Objective response rate (ORR), the primary endpoint, was 9.6% in 115 patients receiving everolimus single agent (stratum 1). Time to tumor progression (TTP) was a secondary endpoint. Interestingly, TTP was 9.7 and 16.7 months in the everolimus single agent and in the everolimus plus octreotide long-acting release (LAR) stratum (stratum 2, *n* = 45), respectively [92]. These results were followed by other two trials of the series. Given the potentially exciting result of the stratum 2 of RADIANT-1, the RADIANT-2 trial was started. It was a randomized phase III trial on everolimus vs. placebo, both added to therapy with octreotide LAR, in advanced functioning NETs. The trial enrolled 429 patients. A median progression-free survival (PFS) of 16.4 and 11.3 months was observed in the everolimus and placebo arm, respectively. The PFS observed in the combination arm confirmed the good results of the RADIANT-1. However, the difference in PFS as assessed by central radiology review was only near-significant, although numerically substantial. The RADIANT-2 hence was a formally negative study. The analysis of the baseline population, furthermore, showed an imbalance in known prognostic characteristics: everolimus arm had a higher proportion of lung primary NETs, moderately differentiated tumors, proportion of previous chemotherapy, higher Chromogranin A and poorer performance status in respect to the placebo arm [93]. The final analysis for overall survival (OS) showed 29.2 vs. 35.2 months in the everolimus and placebo arm, respectively. This difference was not statistically significant. After correction for the baseline imbalances, the difference remained not significant [94]. The combination of everolimus and a SSA has been recently explored in NETs of thoracic origin by the LUNA trial (Table 1). This was the first prospective trial specifically addressed to thoracic NETs, namely lung and thymic. The LUNA trial was a phase II open-label trial in which patients were randomized to receive pasireotide, a SSA with high affinity for SSTR1, 3 and 5, or everolimus or both, in 3 non-comparative arms. The primary end-point, the proportion of progression-free patients at 9 months, was met, deeming the three treatment options as active in the study population. Median PFS for the pasireotide, everolimus and the combination arm was 8.5, 12.5 and 11.8 months, respectively. However, these have to be considered as exploratory results, since it was not the primary end point and there was not a formal comparative analysis [95]. The RADIANT-3 can be considered the natural evolution of the RADIANT-1. This trial was a randomized double-blind placebo-controlled phase III clinical trial evaluating the efficacy of everolimus in panNETs. Four-hundred-and-ten patients affected by low- to intermediate-grade panNET were randomized to receive everolimus or placebo. Crossover was allowed after confirmed progression. A median PFS of 11 vs. 4.6 months was observed in the everolimus and placebo arm, respectively [96]. Final data from the RADIANT-3 trial showed a non-significant difference between the two treatment arms: OS was 44 and 37.7 months in the everolimus and placebo arm, respectively. Since this result was likely affected by the crossover, an exploratory analysis in order to correct for crossover of patients from placebo to everolimus was performed: corrected survival rates were 82% and 75% at 12 months and 67% and 55.6% at 24 months for everolimus and placebo arm, respectively (HR 0.60) [97]. The last act so far of the RADIANT series was the RADIANT-4 trial, which was a phase 3 randomized placebo-controlled clinical trial lead in non-functioning extra-pancreatic NETs. 302 patients were randomized to receive everolimus or placebo. Concomitant SSA treatment was not allowed. Median PFS, the primary endpoint, was 11.0 and 3.9 months in the everolimus and placebo arm, respectively. Preliminary analysis of survival seemed to show a benefit for everolimus over placebo: data were not mature, but the 25th percentile of survival was evaluated to be at 23.7 vs. 16.5 months, respectively [98].

Despite all the efforts made, no clinical and/or pathological biomarker has been identified so far to predict response to everolimus in any of the studies considered.

Based on the results of these trials, everolimus is approved for the treatment of advanced pancreatic, gastro-intestinal and lung NETs.

## 6. Future Perspectives

Over 1300 NET patients were enrolled in the RADIANT trials and a consistent, reproducible median PFS of about 11 months was observed: everolimus, and thus mTOR inhibition, has become a cornerstone in the management of NET patients of pancreatic and extra-pancreatic origin. Researchers are moving towards optimization of patients’ selection and outcomes through combinations and sequences.

There are over 20 active enrolling studies in NETs involving everolimus, alone or in combination with other biologic or chemotherapy agents. There are as much as over 40 studies if we consider active not recruiting studies as well (source: clinicaltrials.gov) [99].

Recently, a phase II study on BEZ235, a mTORC1 and mTORC2 inhibitor, vs. everolimus was conducted in panNETs. The rationale of BEZ235 therapy was to prevent feedback loops via growth factors, such as IGF-1, from activating the pathway through mTORC2 upregulation [18]. The study, nevertheless, was suspended early due to toxicity in the experimental arm. The experience from this trial suggested that extensive inhibition of the PI3K/Akt/mTOR pathway should be carefully evaluated in humans [100]. One of the most clinically interesting designs is the SEQTOR trial one (Table 2). This is an open-label randomized phase III trial in advanced well-differentiated panNETs evaluating the optimal sequence between everolimus followed by streptozotocin and 5-fluorouracile and the opposite one. Primary endpoint is PFS from randomization to second progression of disease (NCT02246127) [101].

Immunotherapy has been a revolution in cancer treatment. However, to date, there is no approved immunotherapy agent in well-differentiated (i.e., G1 and G2 according to WHO 2010 classification) NETs. Conversely, avelumab, an anti-PD-L1 antibody, has been recently approved for the treatment of the Merkel-cell carcinoma following results from a phase II trial [102] and pembrolizumab, an anti-PD-1 antibody, showed promising results in the same disease as well [103]. Results from the many ongoing trials, for example, pembrolizumab in PD-L1-positive well-differentiated NETs (Keynote-028, NCT02054806) [104] or PDR001 in well-differentiated unselected thoracic, pancreatic and gastrointestinal NETs and in GEP-NECs (NCT02955069) [105], are eagerly awaited. Recently, the role of PTEN loss, possibly through compromised autophagy, has been advocated as an escape mechanism from immune system by reduction of effective T-cell priming [63]. This supports the idea of combining immunotherapy with PI3K/Akt/mTOR inhibition, as evaluated in an ongoing phase I trial where pembrolizumab is combined with the PI3K-delta inhibitor, INCB050465 (NCT02646748) [106].

The mTOR has a central converging role for many functions of the cell and serves as a sensor for extracellular signals and nutrients in order to regulate cell growth and proliferation. Its complexity grew the more we found about the pathway, and more needs to be unveiled.

mTOR predominant role in NETs tumorigenesis and progression has been demonstrated in preclinical studies and late clinical trials, and in all kinds of NETs. In this scenario, the unmet clinical needs are patients’ selection and development of acquired resistance. A predictive marker of response to everolimus (or to PI3K/Akt/mTOR inhibition in general) would allow treating physicians to prescribe the drug to the patients more likely to benefit from it, avoid toxicities in likely unresponsive patients, with secondary positive effects on health providers economic balance. Still this would be a challenging issue, given the complexity of and cross-talks involved in this pathway. To avoid or delay development of acquired resistance, combined inhibition of targets, by multi-target inhibitors or by combination of drugs, should be investigated, in order to disrupt feedback loops. Lastly, some clues suggest that combination of mTOR inhibition and immunotherapy, for example, in immune check-point inhibitors, could be a promising strategy in cancer and NET treatment.

## Figures and Tables

**Table 1:** Clinical trials on Everolimus in neuroendocrine tumors (NET).

Title (Phase)	Year	Population	Treatments	PFS (HR)	Remarks
**RADIANT-1 (II)**	2010	160 panNET	(I) Everolimus (II) Everolimus + Octreotide LAR	9.7 16.7	No comparison between strata
**RADIANT-2 (III)**	2011	429 mixed (carcinoid syndrome)	Everolimus + Octreotide LAR vs. pbo + Octrotide LAR	16.4 vs. 11.3 (HR: 0.77)	Not significant by central radiology analysis
**RADIANT-3 (III)**	2011	410 panNET	Everolimus vs. pbo	11 vs. 4.6 (HR: 0.35)	40% concomitant SSA
**RADIANT-4 (III)**	2016	302 mixed non-panceratic	Everolimus vs. pbo	11 vs. 3.9 (HR: 0.48)	Concomitant SSA not allowed
**LUNA trial (II)**	2017	124 thoracic (lung thymic)	Pasireotide Everolimus Everolimus + Pasireotide	8.5 12.5 11.8	No comparison among arms

Year: year of publication; pbo: placebo; SSA: somatostatin analog; PFS expressed in months.

**Table 2:** Ongoing clinical trials including NET patients reported in the manuscript.

Study	NCT	Study Design	Treatment
SEQTOR	NCT02246127	Phase III, well differentiated panNET	Everolimus → STZ+5-FU vs. STZ+5-FU → Everlimus
Keynote-028	NCT02054806	Phase I, PD-L1-positive well-differentiated NETs	Pembrolizumab
CPDR001E2201	NCT02955069	Phase II, well-differentiated unselected thoracic, pancreatic and gastrointestinal NETs and GEP-NECs	PDR001 (anti PD-1)
39110-107	NCT02646748	Phase I, advanced solid tumors	Pembrolizumab + INCB050465 (PI3K-delta inhibitor) (Group B)

STZ: Streptozotocin; 5-FU: 5-Fluorouracile.

## Abbreviations

4EBP1	Eukaryotic translation initiation factor 4E binding protein 1
Akt	Protein kinase B
AMPK	Adenosine monophosphate-activated protein kinase
c-KIT	KIT Proto-Oncogene Receptor Tyrosine Kinase
DEPDC5	DEP domain containing 5
EGFR	Epidermal growth factor receptor
eIF4E	Eukaryotic translation initiation factor 4E
ERK1/2	Extracellular-signal-regulated kinase 1/2
EWSR1	Ewing Sarcoma Breakpoint Region 1
FGF	Fibroblast growth factor
FGFR	Fibroblast growth factor receptor 3
GAP	GTPase activating protein
GATOR	GAP activity towards Rag
GTP	Guanosine triphosphate
HIF1a	Hypoxia inducible factor 1alfa
IGF-1	Insulin-like growth factor-1
IGF-1R	Insulin-like growth factor-1 receptor
IHC	Immunohistochemistry
MAPK	Mitogen-activated protein kinase
mTOR	Mechanistic target of rapamycin
NET	Neuroendocrine tumor
NET	Neuroendocrine tumor
ORR	Objective response rate
OS	Overall Survival
PDGFR	Platelet-derived growth factor receptor
PDK1	3-phosphoinositide-dependent kinase-1
PFS	Progression-free survival
PI3K	Phosphoinositide-3-kinase
PIP2	Phospatidylinositol-4,5,-bisphospate
PIP3	Phospatidylinositol-3,4,5,-trisphospate
PKB	Protein kinase B
PKCa	Protein kinase C-alfa
PPAR-y	Peroxisome proliferator-activated recetpor y
PSPN	Persephin
PTEN	Phosphatase and tensin homolog deleted on chromosome 10
Rag	Ras-related GTPase
Raptor	Regulatory-associated protein of mammalian target of rapamycin
RET	Rearranged during transfection
Rheb	Ras homolog enriched in brain
Rictor	Rapamycin-insensitive companion of mammalian target of rapamycin
RSK1	Ribosomal S6 kinase
S6K1	S6 kinase 1
SGK1	Serum- and glucocorticoid-induced protein kinase 1
SREBP1/2	Sterol regulatory element-binding protein 1/2
SSA	Somatostatin analog
SSTR1-5	Somatostatin receptor 1-5
SSTR1-5	Somatostatin receptor 1-5
TNFa	Tumor necrosis factor alfa
TSC	Tuberous sclerosis
TTP	Time to tumor progression
VEGF	Vascular endothelial growth factor
Wnt	Wingless-related integration site
